# Bilateral Lateral Thoracic Meningoceles: An Unusual Cause of Recurrent Respiratory Infections in a Non‐Syndromic Infant

**DOI:** 10.1002/ccr3.71046

**Published:** 2025-10-08

**Authors:** Rizwan Ali, Rehmat Farhaj, Muhammad Hamza, Fazeela Bibi, Khalil El Abdi, Kinza Masroor Khanzada, Sadia Shafique, Ikhtiar Alam, Marium Mansoor, Roshan Nadeem, Kajal Fnu, Said Hamid Sadat

**Affiliations:** ^1^ Bannu Medical College Bannu Pakistan; ^2^ Saidu Medical College Swat Pakistan; ^3^ Jinnah Medical and Dental College Karachi Pakistan; ^4^ Faculty of Medicine and Pharmacy of Rabat, Mohammed V University Rabat Morocco; ^5^ United Medical and Dental College Karachi Pakistan; ^6^ Lady Reading Hospital Peshawar Pakistan; ^7^ Pak International Medical College Peshawar Pakistan; ^8^ Fatima Jinnah Medical University Lahore Pakistan; ^9^ Pak Red Crescent Medical College Lahore Pakistan; ^10^ Ghulam Mohammad Medical College Sukkur Pakistan; ^11^ Kabul University of Medical Sciences Abu Ali Ibn Sina Kabul Afghanistan

**Keywords:** infant, lateral meningocele, pediatrics, recurrent respiratory infections, spinal dysraphism, thoracic meningocele

## Abstract

Lateral thoracic meningoceles are rare spinal dysraphisms, often associated with neurofibromatosis type 1 (NF‐1) and typically diagnosed in adults. Their presentation in infancy without syndromic features is exceptional. We report the case of a 6‐month‐old female infant who presented with failure to thrive and recurrent respiratory infections. The neurological examination was normal, and there were no cutaneous stigmata of spinal dysraphism or features of Lehman syndrome. Initial imaging raised suspicion for neuroenteric cysts; however, the definitive diagnosis was established by magnetic resonance imaging (MRI), which revealed bilateral lateral meningoceles containing cerebrospinal fluid (CSF) without neural elements. Given the patient's stable neurological status, a conservative management strategy was adopted. The patient was treated for her respiratory symptoms and discharged in stable condition with a plan for close multidisciplinary surveillance. This case highlights an atypical, non‐neurological presentation of a rare congenital spinal anomaly. It underscores the importance of including lateral meningoceles in the differential diagnosis for infants with unexplained recurrent pulmonary symptoms. While surgical excision is indicated for progressive deficits, this report suggests that conservative management is a viable approach for neurologically intact patients.

**Summary**

- Lateral thoracic meningoceles, a rare spinal anomaly, should be considered in the differential diagnosis of infants with unexplained recurrent respiratory symptoms, even without neurological deficits; conservative management is a suitable option for neurologically stable patients.

## 1 Introduction

Lateral meningocele is a rare form of spinal dysraphism where meningeal diverticula herniate through the neural foramina. While most meningoceles are dorsal and lumbosacral, lateral meningoceles typically occur in the thoracic region. The etiology is incompletely understood but is thought to involve failed caudal neural tube closure, influenced by folate deficiency, genetic factors, and environmental exposures [1, 2]. Associations with neurofibromatosis type 1 (NF‐1) and connective tissue disorders like Marfan syndrome have been described [3].

In rare instances, lateral meningoceles are a cardinal feature of Lehman syndrome, an autosomal dominant disorder caused by NOTCH3 gene mutations [4]. This syndrome's phenotype includes distinct craniofacial dysmorphisms, growth deficiency, congenital heart defects, and feeding difficulties [2, 5]. While cognitive impairment and hearing loss have been documented, fewer than half of the 22 reported patients with Lehman syndrome had confirmed loss‐of‐function NOTCH3 mutations [5, 6, 7].

Here, we report a unique case of bilateral thoracic lateral meningoceles in a 6‐month‐old infant who presented without clinically apparent syndromic features. This case highlights the importance of including rare spinal anomalies in the differential diagnosis for infants with unexplained recurrent chest infections.

## 2 Case Description

A 6‐month‐old female infant presented to our tertiary care center for evaluation of failure to thrive and recurrent respiratory infections. She was born at term via spontaneous vaginal delivery following an unremarkable pregnancy with adequate maternal folate supplementation. No family history of genetic disorders was reported. Her medical history was significant for a severe episode of pneumonia at 3 months of age, and she subsequently experienced recurrent chest infections.

On physical examination, the patient was underweight for her age at 5.9 kg. However, her neurological assessment was unremarkable, revealing age‐appropriate tone, reflexes, and intact cranial nerves. No cutaneous stigmata of spinal dysraphism were observed.

## 3 Diagnostics and Follow‐Up

**FIGURE 1:**
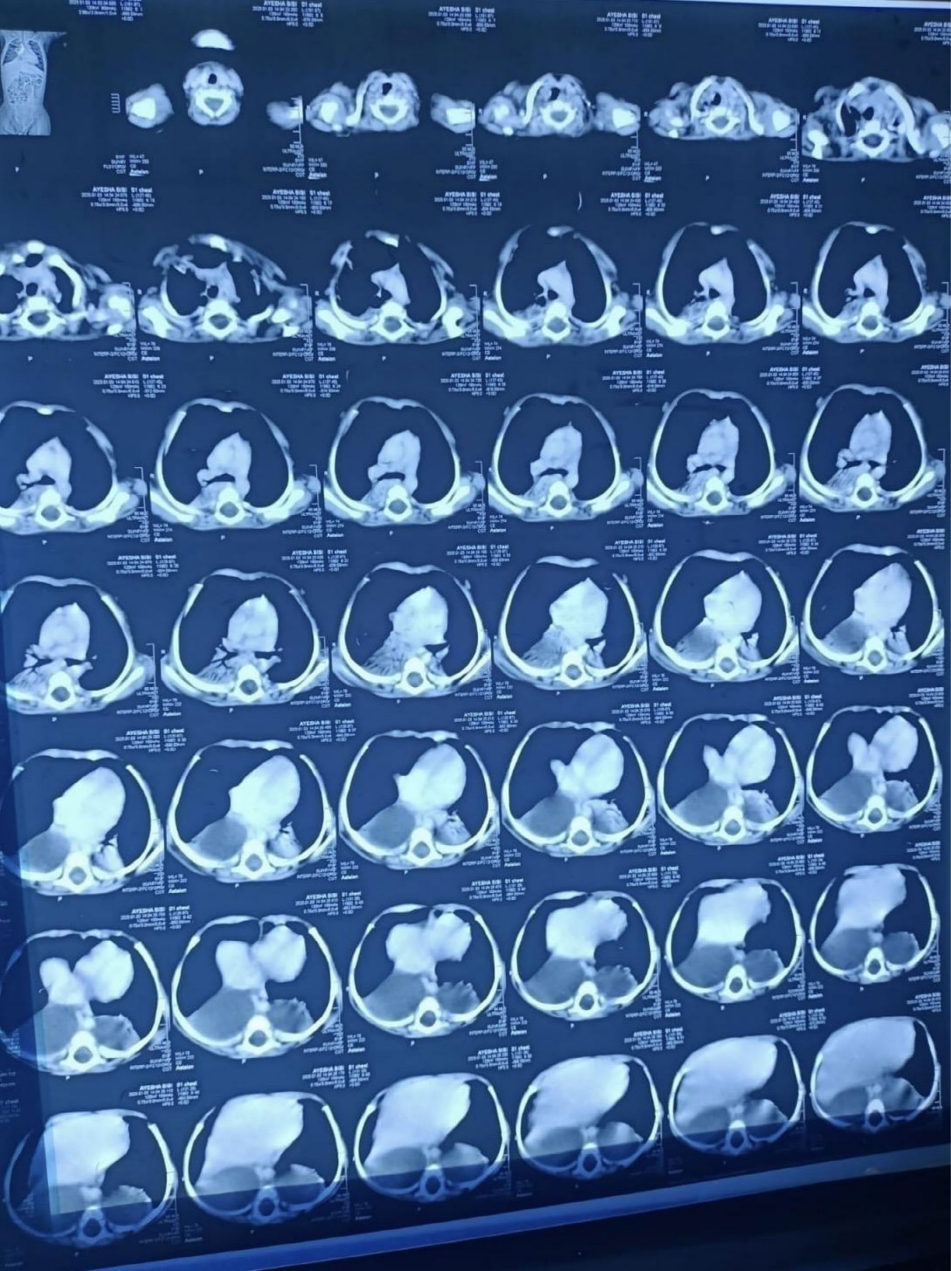
High‐resolution computed tomography (HRCT) of the chest in a 6‐month‐old infant. The axial view demonstrates large, bilateral paravertebral cystic masses, with the right lesion measuring 5.3 × 4.5 cm and the left 4.0 × 4.0 cm. The scan also reveals significant widening of the spinal canal with associated posterior scalloping of the vertebral bodies, findings that initially raised suspicion for neuroenteric cysts.

**FIGURE 2:**
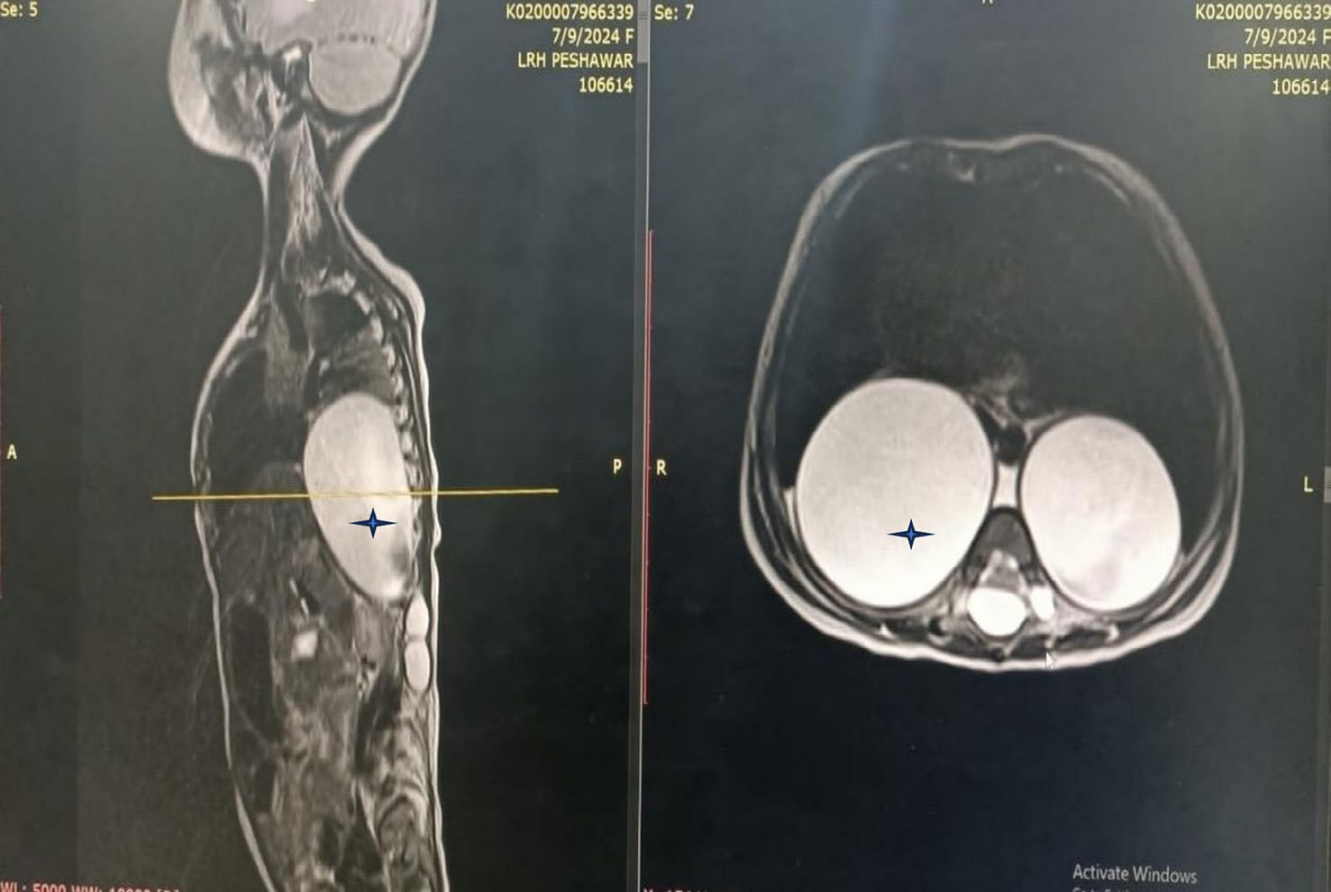
Magnetic resonance imaging (MRI) of the thoracic spine. These T2‐weighted images provide the definitive diagnosis of bilateral lateral meningoceles. The images show large cerebrospinal fluid (CSF)‐filled sacs (hyperintense signal, marked by asterisks) herniating through enlarged neural foramina. The absence of entrapped neural elements within the sacs is clearly demonstrated, confirming severe dural ectasia.

The diagnostic investigation began with thoracic ultrasonography, which identified bilateral paraspinal cystic lesions: a 4.6 × 4.4 cm cyst in the right lower chest and a 3.6 × 3.4 cm cyst in the left, along with mild pleural effusions. A subsequent high‐resolution computed tomography (HRCT) scan provided more detail, measuring the right‐sided lesion at 5.3 × 4.5 cm and the left at 4.0 × 4.0 cm. The HRCT revealed resultant basal consolidation and showed long‐segment widening of the cervico‐dorsal spinal canal with posterior vertebral scalloping (Figure 1), raising suspicion for neuroenteric cysts. For definitive diagnosis, magnetic resonance imaging (MRI) was performed. The MRI established the diagnosis of bilateral lateral meningoceles extending from T6 to L2 (Figure 2). It confirmed that multiple cerebrospinal fluid (CSF)‐filled sacs were herniating through widened neural foramina into the posterior mediastinum, without evidence of entrapped neural elements. A diagnostic aspiration yielded acellular fluid consistent with CSF, confirming the contents. Given the patient's stable neurological status, a conservative management strategy was adopted, focusing on antibiotic therapy for respiratory symptoms. The infant was discharged with a plan for close multidisciplinary surveillance.

## 4 Discussion

Lateral thoracic meningoceles, which are herniations of the dura and arachnoid mater through an enlarged intervertebral foramen, are rare congenital anomalies [8]. First described in 1977 [9], the condition is typically diagnosed in adults and is often associated with neurofibromatosis type 1 (NF‐1) [10]. This case is highly unusual, presenting in a 6‐month‐old infant with an isolated, non‐syndromic bilateral presentation dominated by recurrent respiratory infections rather than the classic symptoms of back pain or paraparesis seen in adults [11, 12].

This atypical presentation required a rigorous differential diagnosis, for which magnetic resonance imaging (MRI) was definitive. While initial imaging suggested neuroenteric cysts, MRI was crucial in differentiating the diagnosis. Neuroenteric cysts are frequently associated with vertebral body segmentation anomalies (e.g., hemivertebrae), which were absent in this case. Furthermore, their signal intensity can be variable due to proteinaceous content, whereas the lesions here demonstrated pure cerebrospinal fluid (CSF) isointensity on all sequences. The definitive feature was the clear, wide‐necked communication between the paraspinal sacs and the thecal sac, a pathognomonic finding for meningoceles that is not characteristic of neuroenteric cysts [13, 14]. The lesions demonstrated pure cerebrospinal fluid (CSF) isointensity, a wide‐necked communication with the thecal sac, and vertebral body scalloping—features pathognomonic for a meningocele rather than an extradural arachnoid cyst [15, 16]. Furthermore, the absence of any solid, enhancing components ruled out cystic neoplasms, and the congenital nature excluded a post‐traumatic pseudomeningocele [14, 17, 18].

With the diagnosis established, the management rationale was clear. Surgical excision is indicated for progressive neurologic deficit or significant respiratory distress [11, 19]. However, as our patient was neurologically intact and clinically stable, a conservative management strategy with close multidisciplinary surveillance was the most appropriate course of action. This surveillance protocol included predefined triggers for re‐evaluation and potential surgical intervention, stratified into clinical and radiological criteria. Clinically, any new or progressive neurological deficit, such as changes in lower limb tone or reflexes, would prompt immediate surgical consultation. Worsening respiratory function, including an increased frequency of infections or signs of respiratory distress attributable to the mass effect of the meningoceles, would also serve as a strong indication. Radiologically, surgical intervention would be considered upon evidence of significant interval growth of the meningoceles, the development of spinal cord compression, or the formation of a syrinx on serial MRI [20]. This case suggests that for neurologically intact infants with atypical presentations, a watchful waiting approach is a viable initial pathway.

## 5 Conclusion

Lateral thoracic meningoceles are rare spinal dysraphisms, typically diagnosed in adults and frequently associated with neurofibromatosis type 1 (NF‐1). This report presents a clinically significant case of bilateral, non‐syndromic lateral meningoceles in a 6‐month‐old infant whose presentation was dominated by recurrent respiratory infections rather than neurological deficits. Definitive diagnosis was established with magnetic resonance imaging (MRI), which characterized the cerebrospinal fluid (CSF)‐filled lesions and confirmed the absence of neural elements. Notably, the patient's stable neurological status permitted a conservative management strategy, deferring surgical intervention. This case underscores the importance of including rare spinal anomalies in the differential diagnosis for infants with unexplained pulmonary symptoms, even without cutaneous stigmata or syndromic features. While limited by its single‐case nature, this report suggests that for neurologically intact infants, a watchful waiting approach is a viable initial pathway, though further reporting is needed to delineate the natural history and long‐term outcomes of such early, atypical presentations.

## Author Contributions

**Rizwan Ali:** conceptualization, data curation, formal analysis, investigation, methodology, project administration. **Rehmat Farhaj:** conceptualization, data curation, investigation, methodology. **Muhammad Hamza:** conceptualization, formal analysis, investigation, methodology, resources. **Fazeela Bibi:** conceptualization, data curation, formal analysis, investigation, methodology. **Khalil El Abdi:** conceptualization, data curation, investigation, methodology, project administration, writing – original draft, writing – review and editing. **Kinza Masroor Khanzada:** methodology, project administration, writing – original draft. **Sadia Shafique:** data curation, formal analysis, investigation. **Ikhtiar Alam:** validation, writing – original draft, writing – review and editing. **Marium Mansoor:** investigation, methodology, writing – original draft. **Roshan Nadeem:** supervision, validation, visualization, writing – original draft. **Kajal Fnu:** methodology, supervision, validation, visualization, writing – original draft. **Said Hamid Sadat:** methodology, project administration, writing – review and editing.

## Ethics Statement

The authors have nothing to report.

## Consent

This case report was conducted in accordance with the ethical principles of the Declaration of Helsinki. Written informed consent was obtained from the patient for publication of this case report and any accompanying images, and all patient data has been anonymized to protect privacy.

## Conflicts of Interest

The authors declare no conflicts of interest.

## Data Availability

The data that supports the findings of this study are available in the Supporting Information of this article.
